# Syndemic Challenges: Addressing the Resurgence of Mpox Amidst Concurrent Outbreaks in the DRC

**DOI:** 10.1155/tbed/1962224

**Published:** 2024-11-20

**Authors:** Olaniyi Abideen Adigun, Olalekan John Okesanya, Mohamed Mustaf Ahmed, Bonaventure Michael Ukoaka, Don Eliseo Lucero-Prisno, Emmanuella Ogechi Onyeaghala, Emmanuel Ayomide Oluwasusi, Olamide Esther Ogunwale, Ayodeji Amos Faniyi

**Affiliations:** ^1^Department of Medical Laboratory Science, Nigerian Defence Academy, Kaduna, Kaduna State, Nigeria; ^2^Department of Medical Laboratory Science, University College Hospital, Ibadan, Oyo State, Nigeria; ^3^Department of Public Health and Maritime Transport, University of Thessaly, Volos, Greece; ^4^Faculty of Medicine and Health Sciences, SIMAD University, Mogadishu, Somalia; ^5^Department of Internal Medicine, Asokoro District Hospital, Abuja, Nigeria; ^6^Department of Global Health and Development, London School of Hygiene and Tropical Medicine, London, UK; ^7^Research and Development Office, Biliran Province State University, Naval, Philippines; ^8^Research and Innovation Office, Southern Leyte State University, Sogod, Philippines; ^9^Department of Medical Laboratory Science, Achievers University Owo, Owo, Ondo State, Nigeria; ^10^Department of Medical Laboratory Science, Ladoke Akintola University of Technology, Ogbomosho, Nigeria; ^11^Department of Medical Laboratory Science, Joseph Ayo Babalola University, Ikeji Arakeji, Nigeria

**Keywords:** anthrax, cholera, Democratic Republic of Congo, epidemiology, measles, monkeypox, mpox, plague, syndemic

## Abstract

The Democratic Republic of Congo (DRC) faces a syndemic of infectious diseases, including monkeypox (mpox), cholera, measles, anthrax, and plague, worsening public health challenges and socioeconomic disparities. This review synthesizes and discusses epidemiological data and consequences of simultaneous outbreaks in the DRC between January 2023 and March 2024. The findings highlight a 6.7% fatality rate and 3319 confirmed cases of mpox, with significant outbreaks in Kinshasa and 22 other provinces. Anthrax occasionally surfaced among cattle-raising villages, measles affected fewer than five children susceptible to the disease, and cholera outbreaks persisted in North Kivu, South Kivu, and Tanganyika. Plague incidences, mostly bubonic, have been reported in Ituri province. Vulnerable groups, including children, mothers, the elderly, and those with compromised immune systems, face increased risks due to poor healthcare access, hunger, and underlying medical conditions. Cultural beliefs, healthcare system issues, and socioeconomic instability impede effective response tactics. This strain on the fragile healthcare system highlights the need for increased surveillance, immunization efforts, and community involvement. To mitigate the effects of syndemic outbreaks, strengthening the DRC's health systems through international cooperation, integrated public health initiatives, and improved access to healthcare is crucial.

## 1. Introduction

The term “syndemic,” which is a combination of the words “synergy” and “epidemic,” denotes a novel approach to comprehending the intricacy of disease interactions among populations. It is defined by three key ideas: illness aggregation, disease interaction, and the general socioeconomic elements that contribute to it. When several coexisting diseases interact, the existence of one disease worsens the severity or prevalence of others. This frequently occurs due to social, economic, and environmental factors [1]. The concept of syndemic challenges is especially important in the Democratic Republic of Congo (DRC), which is afflicted by endemic poverty, political instability, and a weak healthcare system. The DRC is facing several health crises, with the resurgence of monkeypox (mpox) serving as a focal point amidst epidemics of cholera, measles, anthrax, and plague [1].

Mpox is an enveloped double-stranded DNA virus that belongs to the *Orthopoxvirus* genus of the Poxviridae family. Recent genomic analyses have provided insights into the evolution and spread of viruses. Studies have identified two distinct clades: clade I (formerly the Congo Basin clade) and clade II (formerly the West African clade). The 2022 global outbreak has been attributed to clade II (formerly the West African clade), which shows evidence of ongoing human-to-human transmission and adaptation to human hosts [2, 3]. The virus was first isolated in 1958 in a Danish laboratory from the skin lesions of an imported macaque and is known to cause epidemics in captive primates [4]. The first human case of mpox was reported in 1970 in the DRC. Since then, several human cases of mpox have been reported in the tropical rainforests of Western and Central African countries. The most common form of transmission is through contact with wild animals such as rodents. Mpox is widespread in densely wooded areas of West, Central, and East Africa, particularly in northern and central regions [5]. Eleven of the DRC's 26 provinces are endemic for mpox, although in recent times the overall number of mpox cases and provinces reporting mpox has increased to 22 as of November 2023 [5]. Since 2022, there has been a global epidemic of clade IIb mpox, affecting several nations outside Africa that have not previously encountered mpox. The DRC did not report any cases of mpox associated with clade IIb during the worldwide outbreak, and only mpox clade I was discovered in the country [6]. Prior to April 2023, there were no formally documented cases of clade I mpox sexual transmission worldwide. The first known instances were reported when a Belgian man with connections to the DRC tested positive for clade I during a visit to Kenge, Kwango Province [7]. Subsequently, sexual interactions with this patient in the DRC tested positive for clade I mpox, which contains viral sequences that are closely related. Another outbreak has been reported in Brazil, with several instances of mpox among sex workers [5]. The dynamics of mpox clade I transmission in the DRC are poorly understood due to a lack of timely access to diagnostics, difficulties in linking cases to any contact with infectious animals, and insufficient epidemiological and contact tracing investigations over time. These new sexual and undiscovered mechanisms of transmission have raised concerns regarding the spread of the outbreak in the country [7].

The current health crisis in the DRC is characterized by a convergence of multiple infectious disease outbreaks, each presenting unique challenges to public health. Cholera outbreaks, fueled by inadequate access to clean water and sanitation facilities, continue to afflict communities across the country, exacerbating the burden on already strained healthcare systems [8]. Measles outbreaks, exacerbated by suboptimal vaccination coverage and weakened healthcare infrastructure, pose significant threats to vulnerable populations, particularly to children. Additionally, outbreaks of anthrax and plague further compounded the health crisis in the DRC, highlighting the multifaceted nature of the syndemic challenges. Anthrax, a bacterial infection typically transmitted through contact with contaminated animal products, presents challenges in rural areas, where livestock rearing is prevalent. Plague, caused by the bacterium *Yersinia pestis* and transmitted through fleas, poses risks of epidemic spread, particularly in regions with poor sanitation and overcrowded living conditions [9]. Against this backdrop of concurrent outbreaks, the resurgence of mpox adds a layer of complexity to the health crisis in the DRC, particularly in resource-constrained settings [8]. This review aims to address the gap in our understanding of mpox transmission dynamics, particularly the new sexual transmission pathways and their public health implications. By exploring concurrent outbreaks of cholera, measles, anthrax, and plague alongside mpox, it seeks to highlight the compounded challenges faced by the healthcare system in the DRC. Additionally, the review discusses broader impacts on public health and provides recommendations for improving response strategies and healthcare resilience in the face of these multifaceted crises. This synthesis contributes new insights into the interactions between these infectious diseases and their impact on the DRC's health landscape, offering a foundation for more effective interventions and policy responses.

### 1.1. Epidemiology and Burden of Mpox Amidst Multiple Outbreaks

In August 2023, mpox cases were first confirmed in Kinshasa, the capital of the DRC. There were four separate incidents in which individuals exposed in other provinces (Equateur and Mai-Ndombe) traveled to Kinshasa, leading to local transmission and small clusters of cases in the capital. Between August 18 and November 12, 2023, 102 suspected cases were reported across eight health zones in Kinshasa. This included 18 confirmed cases and one confirmed death from mpox, resulting in a case fatality ratio of 5.6% among confirmed cases [7]. The initial confirmed mpox case was a person who traveled by riverboat from Mai-Ndombe province, where mpox is endemic to Kinshasa. This case was confirmed on August 18. Subsequently, several close contacts showed symptoms and tested positive for mpox. Additional confirmed cases have been reported in Limete, Makala, and Nsele health zones. Among the confirmed cases in Kinshasa, the sex ratio was two men for every woman, with a median age of 24 years (95% confidence interval [CI] 11–27 years). Currently, 13 confirmed cases, including those of health workers, have recovered. One patient died, and four were still isolated and under treatment. The deceased individuals also had tuberculosis and contracted mpox during hospitalization, indicating a possible nosocomial transmission [7].

**Figure 1:**
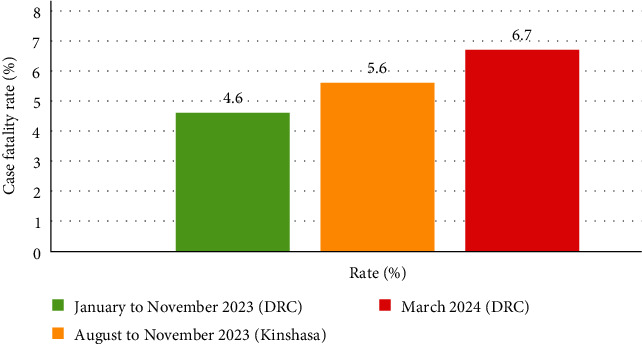
Comparison of mpox case fatality rates over different periods. DRC, Democratic Republic of Congo; mpox, monkeypox.

Between January 1 and November 12, 2023, 12,569 suspected mpox cases and 581 suspected deaths (case fatality rate: 4.6%) were reported across 156 health zones in 22 of the 26 provinces (85%) in the DRC. This marks the highest annual number of cases recorded, with new cases emerging in previously unaffected areas, such as Kinshasa, Lualaba, and South Kivu (Figure 1). Among the suspected cases, 1106 were tested using real-time polymerase chain reaction (RT-PCR), and 714 were confirmed positive for mpox (65% positivity rate) [7].

**Figure 2:**
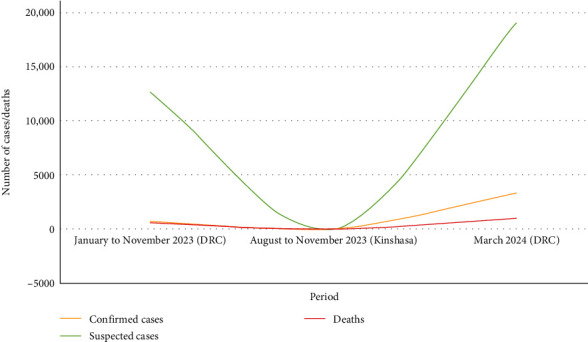
Trends in mpox cases and deaths over time. DRC, Democratic Republic of Congo; mpox, monkeypox.

There has been a noticeable increase in the number of mpox cases reported in the DRC [9, 10]. Since the release of the Threat Assessment Brief titled “Implications for the EU/EEA of the outbreak of mpox caused by mpox virus clade I in DR Congo in December 2023,” awareness and tracking of the disease have intensified, highlighting the urgent need for preventive measures and response strategies [11]. As of March 29, 2024, 1007 people had died, and 18,922 suspected cases of mpox had been reported to the DRC. As of March 29, 2024, 3319 of 4488 reported cases had been confirmed (Figure 2). In 2024, there were 279 documented fatalities in the nation, translating to a 6.7% fatality rate across 23/26 provinces in the DRC [12].

Simultaneously, the DRC faced outbreaks of other infectious diseases. Cholera continues to be a significant public health challenge, with major outbreaks reported in the North, South, and Tanganyika Provinces [13]. Measles outbreaks persist, particularly in children under five, with suboptimal vaccination coverage contributing to continued transmission [14]. Sporadic anthrax outbreaks have been reported, which mainly affect livestock and rural populations. Additionally, plague cases, primarily the bubonic form, have been periodically reported in the Ituri Province, necessitating ongoing surveillance and rapid response efforts [15, 16].

The burden of mpox and other concurrent outbreaks disproportionately affects vulnerable populations including children, mothers, teenage girls, the elderly, and immunocompromised individuals (Table 1). These groups face higher risks due to their limited access to healthcare, nutritional deficiencies, and underlying health conditions [17]. For instance, children under 5 years are particularly susceptible to severe outcomes from mpox and measles due to their developing immune systems and low vaccination rates [17]. Mothers and teenage girls often bear the brunt of care responsibilities, making them more likely to be exposed to infectious diseases within their households and communities. Elderly individuals, who may have comorbidities such as tuberculosis, are at an increased risk of severe complications and mortality from mpox. Immunocompromised individuals, including those living with human immunodeficiency virus (HIV)/acquired immunodeficiency syndrome (AIDS), face heightened vulnerability due to their weakened immune systems, leading to more severe disease progression and increased mortality rates [18]. Men who have sex with men may face stigmatization and barriers to accessing healthcare, exacerbating the risk of severe outcomes [19, 20]. Refugees and asylum seekers, who often live in crowded and unsanitary conditions, are particularly vulnerable to infectious disease outbreaks. Their mobility and instability in living situations make it challenging to ensure adequate vaccination and healthcare access [21]. The compounded burden of multiple outbreaks strains the already fragile healthcare system in the DRC, limiting its capacity to provide adequate care and preventive measures to vulnerable groups. This situation highlights the urgent need for targeted public health interventions, including vaccination campaigns, improved healthcare access, and comprehensive surveillance systems, to protect high-risk populations and mitigate the impact of ongoing and future outbreaks.

### 1.2. Challenges of Mpox Amidst the Syndemic

The healthcare system in the DRC is severely underdeveloped and underresourced, with a ratio of 0.1 doctors per 1000 people, which is far below the World Health Organization's (WHO's) recommended minimum of one doctor per 1000 people [22]. Access to medical care is extremely limited, particularly in rural and conflict-affected areas. This shortage of healthcare professionals and facilities means that the early detection, diagnosis, and treatment of mpox cases are often delayed, allowing the disease to spread widely [23]. Moreover, the vast and dense tropical forests of the DRC serve as natural reservoirs for the mpox virus. Human encroachment into forested areas, coupled with hunting and consumption of wild animals, increases the risk of zoonotic transmission of the virus. Additionally, the remote and inaccessible nature of many areas complicates efforts to conduct effective surveillance, deliver healthcare, and implement vaccination campaigns [24]. These challenges are compounded by the high levels of poverty, malnutrition, and limited access to clean water and sanitation facilities, which are prevalent in the DRC. These conditions contribute to a weakened population, which is susceptible to infectious diseases. Moreover, socioeconomic instability often forces people to rely on bushmeat for sustenance, thereby increasing the risk of exposure to zoonotic infections [25, 26].

Ongoing conflicts and insurgency in various parts of the DRC have further disrupted public health efforts and led to population displacement. Internally displaced persons (IDPs) often live in overcrowded and unsanitary conditions, which facilitate the spread of infectious diseases. Conflict zones are typically inaccessible to health workers, making it challenging to conduct surveillance, provide medical care, and implement control measures [27]. Cultural beliefs and practices can also hinder disease control. There is often skepticism and mistrust toward government health initiatives and vaccines. Misunderstanding and misinformation about mpox can lead to resistance to preventive measures, including vaccination and the isolation of infected individuals [10, 28]. The DRC surveillance and reporting systems for infectious diseases are inadequate. Many cases of mpox have not been reported because of the lack of diagnostic facilities and trained personnel capable of accurately identifying the virus. Mpox is often misdiagnosed as febrile illness or skin disease. Without accurate data, it is challenging to understand the true scale of an outbreak and to effectively allocate resources [29, 30]. This situation has been exacerbated by a significant shortage of vaccines and antiviral treatments for mpox in the DRC. The existing vaccines are primarily smallpox vaccines that offer protection against mpox; however, their availability is limited. This scarcity is compounded by logistical challenges in distributing vaccines to remote and conflict-affected areas [29]. Environmental factors, such as deforestation and habitat destruction due to logging, mining, and agricultural expansion, increase human–wildlife interactions and increase the risk of zoonotic diseases such as mpox spilling over into human populations. Environmental degradation also disrupts ecosystems, potentially altering the dynamics of disease reservoirs and vectors [30].

The disease spreads more easily in socioeconomically disadvantaged groups because of vulnerability caused by poor immunization rates [31]. The spread of other infectious diseases further complicates this situation [32]. Cholera outbreaks driven by inadequate access to clean water and sanitation are particularly rare in rural and impoverished areas [33]. Measles outbreaks are exacerbated by suboptimal vaccination coverage and a weakened healthcare infrastructure. Population displacement due to conflicts leads to overcrowding and unsanitary conditions, facilitating the spread of diseases, such as measles. Anthrax presents significant challenges in rural areas where livestock rearing is prevalent, and environmental degradation increases the risk of zoonotic transmission. The plague poses epidemic risks in regions with poor sanitation and overcrowded living conditions [34]. The difficulty in treating plague cases is compounded by a lack of access to effective antibiotics and the emergence of antimicrobial-resistant strains of pathogens, similar to the challenges faced with mpox. Complicating this issue is the emergence of antimicrobial-resistant strains of pathogens, including those related to mpox, which represent a significant barrier to outbreak control. The difficulty in treating mpox cases is compounded by the lack of access to effective antibiotics and indiscriminate use of antimicrobial medications [34].

### 1.3. Diagnosis, Treatment, and Prevention of Mpox

Patients with symptoms such as a characteristic rash that progresses through macules, papules, vesicles, pustules, and scabs, along with fever, headache, muscle aches, backache, swollen lymph nodes, chills, and exhaustion should be suspected of having mpox [35]. A history of recent travel to endemic areas, contact with wild animals, particularly rodents and primates, or close contact with infected individuals further supports the suspicion of mpox [36]. Mpox virus in skin lesions, blood, or respiratory secretions can be confirmed by laboratory testing. Before initiating antiviral treatment, lesion samples should be collected and promptly sent to the laboratory [24, 37]. The PCR is the primary method used to detect mpox DNA. Although it is sensitive and specific, PCR is not widely available [37]. Virus isolation through culture and electron microscopy and serological tests to detect specific antibodies (immunoglobulin M [IgM] and immunoglobulin G [IgG]) are additional diagnostic methods used in laboratories [38].

Antiviral therapy has been shown to reduce mortality and morbidity by mitigating symptom severity. Although controlled trial data are limited, studies have indicated that early antiviral administration reduces the severity and duration of illness [39, 40]. For instance, one study found that tecovirimat (TPOXX) was effective in reducing symptoms and duration when administered promptly [41]. The preferred method of administration is oral or intravenous, depending on the severity of the case, to achieve therapeutic blood levels quickly. The extent of local lesions and the duration of illness influenced the recommended antiviral dosage. It is significant to note that antivirals do not affect the virus that has already entered host cells [42]. The main treatment for mpox includes supportive care and antivirals, such as TPOXX, which should be started immediately to mitigate symptoms [43]. Early antiviral therapy reduces viral replication, hastens patient recovery, and prevents the spread of the infection to others. Alternative options, such as cidofovir or brincidofovir, can be considered in severe cases or in immunocompromised patients. If skin lesions are present, treatment involves comprehensive wound care to prevent secondary bacterial infections [44, 45].

The main method of preventing mpox infection is vaccination with mpox or smallpox. Combination vaccines such as JYNNEOS (Imvamune or Imvanex) have been approved to prevent both mpox and smallpox. ACAM2000, a live attenuated vaccine for smallpox, can also provide cross-protection against mpox, although it is used less frequently owing to its higher risk profile [46]. Public health measures such as surveillance, rapid identification, and isolation of infected individuals are crucial for preventing outbreaks. Quarantine measures for infected patients and close contacts can help control the spread of the virus [47]. Personal protective equipment (PPE), including gloves, masks, and protective clothing, should be used by healthcare workers and caregivers. Regular hand washing with soap and water, or the use of alcohol-based hand sanitizers, is essential. Avoiding contact with potentially infected animals and ensuring safe handling and decontamination of materials that may be contaminated with the virus are also important preventive practices [48]. Public awareness campaigns are necessary to inform the public about the risks, symptoms, and preventive measures against mpox [49]. Training healthcare workers to recognize, diagnose, and manage mpox cases is equally important. Vaccination schedules may vary depending on the epidemiology and resources available in the different regions (Table 2). In endemic areas, vaccination strategies may include both pre-exposure prophylaxis (PrEP) for high-risk individuals and postexposure prophylaxis (PEP) for those who have been exposed to mpox [46, 50].

### 1.4. Public Health Implications of the Syndemic Challenges

Mpox can cause significant public health concerns due to its potential to cause outbreaks, particularly in regions where the virus is endemic. Effective containment requires robust public health infrastructure [51]. During epidemics, healthcare systems face increased strain due to the need for isolation, treatment of infected patients, and implementation of preventive measures. Mpox outbreaks can lead to economic losses due to healthcare costs, quarantine measures, and disruptions in trade and travel, particularly in the affected regions. Infected individuals may face stigma and discrimination, which affect their mental health and social interactions [31, 52]. The threat of mpox can cause widespread fear and anxiety in the population, leading to behavioral changes and societal disruptions [53]. Complications such as rash caused by mpox can result in permanent scarring, particularly on the face and hands, affecting the patient's appearance and potentially leading to psychological distress [2].

Open skin lesions can become infected with bacteria, leading to complications, such as cellulitis, abscesses, and sepsis. Mpox can lead to viral pneumonia, particularly in immunocompromised individuals who require intensive medical intervention. In rare cases, the virus can cause inflammation of the brain, leading to encephalitis [54], which can result in long-term neurological deficits or death. Severe infections can provoke seizures, which necessitate careful monitoring and management. The virus can also infect the eyes, leading to keratitis (inflammation of the cornea) [55, 56] and conjunctivitis (inflammation of the conjunctiva), resulting in discomfort, potential visual impairment, or vision loss. Some patients may experience chronic pain at the site of the skin lesions or complications from the disease [55]. The psychological impact of mpox, including anxiety, depression, and post-traumatic stress disorder (PTSD), can persist long after the physical symptoms have resolved [53]. Although mpox generally has a lower fatality rate than smallpox, severe cases can lead to death, particularly in young children, pregnant women, and immunocompromised individuals. The case fatality rate can vary based on the strain of the virus and the patient's overall health [57].

Cholera outbreaks in the DRC disproportionately affect vulnerable populations, particularly children with low immunity, who are susceptible to severe dehydration and mortality from cholera, and also disrupt social cohesion and community resilience, exacerbate socioeconomic disparities, and marginalize vulnerable populations. Females, especially those responsible for caregiving, face increased exposure to contaminated water sources during their daily activities, exacerbating their risk of infection [32]. Elderly individuals, with weakened immune systems and limited access to healthcare, experience higher mortality rates during cholera outbreaks. Individuals living with HIV/AIDS are at heightened risk due to compromised immunity, necessitating integrated healthcare services to manage both conditions concurrently [8]. Measures primarily affect children under five, which is exacerbated by suboptimal vaccination coverage and logistical barriers in remote areas. Children face severe complications, such as pneumonia and encephalitis, underscoring the critical need for robust vaccination campaigns and outreach efforts. Females, particularly mothers and caregivers, bear the burden of caring for sick children, often at the expense of their health. Elderly individuals with underlying health conditions are at an increased risk of developing severe measles complications. Refugees and asylum seekers residing in overcrowded and unsanitary conditions face challenges in accessing healthcare and vaccination services, further complicating efforts to control measles outbreaks [14, 33]. Anthrax outbreaks in rural areas of the DRC have affected livestock and subsistence farmers, resulting in significant economic losses and food insecurity. Livestock deaths disrupt agricultural livelihoods, particularly affecting vulnerable rural populations that depend on livestock for income and food. The elderly, with limited access to veterinary services, bear the brunt of economic losses from anthrax outbreaks [58, 59]. Periodic reports of bubonic plague in the Ituri province highlight the ongoing threat of this ancient disease to the DRC. Plague outbreaks can spread rapidly in densely populated areas, necessitating robust disease surveillance and rapid response to promote early detection and treatment [60].

The syndemic of mpox and other diseases in the DRC has led to significant strain on the country's healthcare system, resulting in inadequate access to vulnerable populations. Affected populations face barriers to healthcare, leading to delayed treatment and poor health outcomes. Integrated healthcare services are crucial for improving health equity and resilience against syndemic diseases [61]. Syndemic diseases impose substantial financial burdens on the DRC, diverting resources from essential services and infrastructure development. Outbreak response efforts, such as disease surveillance, vaccination campaigns, and healthcare provision, require significant investments. Economic losses from livestock deaths during anthrax outbreaks contribute to poverty and food insecurity in vulnerable rural communities [14]. The social and economic implications of syndemic diseases include the disruption of community dynamics, exacerbating socioeconomic disparities, and marginalizing vulnerable populations. Outbreaks strain community resources and support networks, increase social isolation and dependency, and exacerbate social exclusion and discrimination during health emergencies [62]. Productivity and human capital development are undermined in the DRC, affecting education, employment, and economic opportunities for the vulnerable populations (Table 3). Children, females, and the elderly experience reduced productivity and economic independence [63].

### 1.5. Recommendation

Implementing an integrated disease surveillance and response (IDSR) system to monitor and report mpox, cholera, measles, anthrax, and plaque cases is crucial [29, 48]. This system should include real-time data collection and analysis to enable the rapid identification and response to outbreaks. Training healthcare workers in disease detection, reporting, and management is essential for ensuring accurate and timely data collection. Strengthening laboratory capacity for accurate diagnostics complements these efforts and provides a foundation for effective disease surveillance and response [7, 37]. Thus, the expansion of immunization programs is critical. Mass vaccination campaigns against mpox, measles, and cholera are expedient [46], particularly in high-risk areas and can significantly reduce the disease incidence. Engaging with local communities to increase vaccination coverage and address vaccine hesitancy is necessary [43]. Utilizing community health workers to disseminate information and facilitate vaccination efforts can enhance the effectiveness of these campaigns.

The launch of extensive public health education campaigns will further support such efforts. Informing the population about the symptoms, transmission, and prevention of mpox, cholera, measles, anthrax, and plague through radio, television, social media, and community meetings will increase awareness and promote preventive measures. Implementing behavior change communication (BCC) strategies is essential to promoting hygiene practices, safe water consumption, and behaviors that reduce the risk of infection [64]. Allocating resources to strengthen healthcare facilities is vital. Ensuring that healthcare facilities are equipped to handle multiple outbreaks simultaneously includes providing adequate supplies of medicines, vaccines, PPE, and other essential medical supplies. Increasing the number of trained healthcare professionals, including doctors, nurses, and community health workers, will help manage the increased burden of disease and improve overall healthcare delivery [65].

Forming global health partnerships with international health organizations, including the WHO, Centers for Disease Control and Prevention (CDC), and nongovernmental organizations (NGOs), is essential for collaborative efforts. These partnerships can provide crucial support for outbreak response, resource provision, and capacity building. Advocating increased international funding to support the DRC's health initiatives will focus on strengthening the long-term health system and emergency response capabilities [66]. Demonstrating strong leadership and coordination from the national government is necessary to oversee and integrate efforts across different sectors including health, water and sanitation, education, and transportation. Collaboration between health authorities, NGOs, community leaders, and international partners will create a unified response to the syndemic, ensuring a comprehensive and effective approach to managing these concurrent health threats [67].

### 1.6. Limitations

This review had several limitations. First, our authorship does not include a representative from the DRC, which may limit the depth of the local insights provided. We recognize that including a local expert could have enhanced the relevance of the manuscript and provided valuable context-specific information. Second, it is important to note that the authors were not directly involved in on-ground outbreak response efforts in the DRC. Our analysis was based on published data, reports, and literature from both local and international sources. Although we have made every effort to provide a comprehensive review, the lack of first-hand experience in the DRC's outbreak response may have limited some aspects of our analysis. These limitations highlight the importance of collaborative research and the need for increased engagement with local experts in future studies on this topic.

## 2. Conclusion

Addressing the resurgence of mpox during concurrent outbreaks of cholera, measles, anthrax, and plague in the DRC requires a coordinated public health response to combat mpox and other diseases such as cholera, measles, anthrax, and plague. Strengthening the health system through an IDSR system, vaccination campaigns, public health education, and BCC strategies, as well as increasing healthcare infrastructure and training professionals, is also crucial. Global health partnerships and increased international funding are required for long-term health system support. Strong leadership and collaboration among health authorities, NGOs, community leaders, and international partners will ensure a unified response. Future research on this topic would benefit from direct collaboration with local experts and healthcare professionals in the DRC to provide more nuanced insights into the challenges and opportunities to improve the outbreak response in the region.

## Figures and Tables

**Table 1:** Epidemiological overview of mpox outbreak in the DRC.

Epidemiological indicator	Kinshasa (August to November 2023)	DRC (January to November 2023)	DRC (March 2024)
Confirmed cases	18	714	3319
Suspected cases	102	12,569	18,922
Deaths	1 confirmed	581 (suspected)	1007
Case fatality rate	5.6%	4.6%	6.7%
Provinces affected	Kinshasa	22 of 26	23
Testing	Not specified	1106 (RT-PCR)	4488 (RT-PCR)
Other outbreaks	Cholera, measles, anthrax, and plague	Cholera, measles, anthrax, and plague	—
Vulnerable groups	Children, mothers, elderly, immunocompromised, refugees	Children, mothers, elderly, immunocompromised, refugees	—
Public health challenges	Local transmission, nosocomial transmission, and increased cases in new areas	Highest annual cases recorded, multiple outbreaks	Mpox fatalities, multiple outbreaks

Abbreviations: DRC, Democratic Republic of Congo; mpox, monkeypox; RT-PCR, real-time polymerase chain reaction.

**Table 2:** Disease management and public health strategies.

Disease	Diagnosis	Treatment	Public health prevention strategies
Mpox	Clinical evaluation, PCR for viral DNA, electron microscopy of skin lesions	Supportive care, antiviral medications (e.g., tecovirimat), smallpox vaccines	1. Vaccination programs: strengthening vaccination coverage 2. Improved WASH: enhancing access to clean water and sanitation 3. Surveillance and reporting systems: by establishing robust surveillance systems 4. Health education and community engagement: educate communities and combat misinformation 5. Strengthening healthcare infrastructure: improve access to diagnostics and treatment 6. Vector control and environmental management: implementing vector control and addressing environmental factors 7. Antimicrobial stewardship: promoting rational use of antibiotics 8. Preparedness and response planning: develop preparedness and response plans

Cholera	Isolation of *Vibrio cholerae* from stool samples, rapid diagnostic tests (RDTs)	Oral rehydration therapy (ORT), intravenous rehydration, antibiotics (e.g., doxycycline and azithromycin)	See above

Measles	Clinical symptoms (fever, cough, conjunctivitis, rash), serological tests, PCR for viral RNA	Supportive care, vitamin A supplements, antibiotics for secondary infections	See above

Anthrax	Isolation of *Bacillus anthracis* from blood/skin/respiratory samples, PCR, immunohistochemical staining	Antibiotics (e.g., ciprofloxacin, doxycycline, and penicillin), antitoxins for inhalational anthrax	See above

Plague	Identification of *Yersinia pestis* in blood/sputum/lymph node aspirates, culture, PCR, serological tests	Antibiotics (e.g., streptomycin, gentamicin, and doxycycline), supportive care	See above

Abbreviations: Mpox, monkeypox; PCR, polymerase chain reaction; WASH, water, sanitation, and hygiene.

**Table 3:** Healthcare and public health challenges in the DRC.

Healthcare system challenges in the DRC	Transmission factors	Socioeconomic and environmental factors
Doctors per 1000 people	0.1 (far below WHO recommendation)	High poverty and malnutrition
Access to medical care	Limited, especially in rural and conflict-affected areas	Inadequate sanitation
Mpox transmission	Dense tropical forests, zoonotic transmission from wildlife	Lack of clean water
Impact of conflict and insurgency	Displacement, overcrowding, and unsanitary conditions	Environmental degradation
Cultural and healthcare system challenges	Mistrust in government health initiatives, inadequate surveillance	Limited healthcare access
Vaccine and treatment shortages	Limited availability, logistical challenges	Healthcare system strain
Other concurrent infectious diseases	Cholera, measles, anthrax, plague, and antimicrobial resistance	Infectious disease outbreaks

Abbreviations: DRC, Democratic Republic of Congo; mpox, monkeypox; WHO, World Health Organization.

## Data Availability

Not applicable because no new data or databases were used in the preparation of this work.
